# Characterization of Neutrophil Functional Responses to SARS-CoV-2 Infection in a Translational Feline Model for COVID-19

**DOI:** 10.3390/ijms251810054

**Published:** 2024-09-19

**Authors:** Sachithra Gunasekara, Miruthula Tamil Selvan, Chelsea L. Murphy, Shoroq Shatnawi, Shannon Cowan, Sunil More, Jerry Ritchey, Craig A. Miller, Jennifer M. Rudd

**Affiliations:** 1 Department of Veterinary Pathobiology, College of Veterinary Medicine, Oklahoma State University, Stillwater, OK 74078, USA; sachithra.gunasekara@okstate.edu (S.G.); mtamils1@jh.edu (M.T.S.); shoroq.shatnawi@okstate.edu (S.S.); sunil.more@okstate.edu (S.M.); jerry.ritchey@okstate.edu (J.R.);; 2 Department of Mathematical Sciences, College of Arts and Sciences, Oklahoma State University, Stillwater, OK 74078, USA

**Keywords:** COVID-19, SARS-CoV-2, feline, neutrophil, neutrophil extracellular traps

## Abstract

There is a complex interplay between viral infection and host innate immune response regarding disease severity and outcomes. Neutrophil hyperactivation, including excessive release of neutrophil extracellular traps (NETs), is linked to exacerbated disease in acute COVID-19, notably in hospitalized patients. Delineating protective versus detrimental neutrophil responses is essential to developing targeted COVID-19 therapies and relies on high-quality translational animal models. In this study, we utilize a previously established feline model for COVID-19 to investigate neutrophil dysfunction in which experimentally infected cats develop clinical disease that mimics acute COVID-19. Specific pathogen-free cats were inoculated with SARS-CoV-2 (B.1.617.2; Delta variant) (*n* = 24) or vehicle (*n* = 6). Plasma, bronchoalveolar lavage fluid, and lung tissues were collected at various time points over 12 days post-inoculation. Systematic and temporal evaluation of the kinetics of neutrophil activation was conducted by measuring markers of activation including myeloperoxidase (MPO), neutrophil elastase (NE), and citrullinated histone H3 (citH3) in SARS-CoV-2-infected cats at 4 and 12 days post-inoculation (dpi) and compared to vehicle-inoculated controls. Cytokine profiling supported elevated innate inflammatory responses with specific upregulation of neutrophil activation and NET formation-related markers, namely IL-8, IL-18, CXCL1, and SDF-1, in infected cats. An increase in MPO-DNA complexes and cell-free dsDNA in infected cats compared to vehicle-inoculated was noted and supported by histopathologic severity in respiratory tissues. Immunofluorescence analyses further supported correlation of NET markers with tissue damage, especially 4 dpi. Differential gene expression analyses indicated an upregulation of genes associated with innate immune and neutrophil activation pathways. Transcripts involved in activation and NETosis pathways were upregulated by 4 dpi and downregulated by 12 dpi, suggesting peak activation of neutrophils and NET-associated markers in the early acute stages of infection. Correlation analyses conducted between NET-specific markers and clinical scores as well as histopathologic scores support association between neutrophil activation and disease severity during SARS-CoV-2 infection in this model. Overall, this study emphasizes the effect of neutrophil activation and NET release in SARS-CoV-2 infection in a feline model, prompting further investigation into therapeutic strategies aimed at mitigating excessive innate inflammatory responses in COVID-19.

## 1. Introduction

The emergence of severe acute respiratory syndrome coronavirus 2 (SARS-CoV-2), the causative agent of coronavirus disease 2019 (COVID-19), has stretched the limits of our ability to maintain global health on a scale unparalleled in recent history [1]. As evidenced by the reported mortality of approximately 7 million people as of July 2024 and the continued emergence of new variants and subvariants, the pandemic has exhausted healthcare systems, strained economies, and challenged societies worldwide [1,2]. COVID-19 manifests as a spectrum of clinical presentations, ranging from mild upper respiratory symptoms to severe pneumonia, acute respiratory distress syndrome (ARDS), and multi-organ dysfunction [1,3]. This variability in disease severity accentuates the complexity of host–pathogen interactions and the critical role of host immune responses in determining disease outcomes. The unique host is a decisive factor when explaining disease heterogeneity, infection rates, and long-term medical consequences. While there have been substantial advances in understanding clinical manifestations, prevention, and epidemiology of COVID-19 in people, there is still an urgent need to explore pathogenesis and immune responses more comprehensively [1,4]. The complexity of these immune responses, particularly the role of neutrophils, is increasingly evident when examining the pathogenesis of COVID-19 [5,6].

Neutrophils, the most abundant white blood cells in circulation and sentinel first line defenders, are integral to the innate immune response against microbial pathogens, including SARS-CoV-2 [7,8,9,10]. Rapid recruitment of neutrophils to the site of infection is initiated through the detection of viral components by pattern recognition receptors (PRRs) such as toll-like receptors (TLRs), complement receptors, and other sensing mechanisms. Chemokines and cytokines, such as interleukin-8 (IL-8), released by infected cells and other immune cells facilitate recruitment, highlighting a coordinated immune response aimed at containing viral spread and mitigating tissue damage [8,10,11,12]. Upon arrival at the site of infection, neutrophils deploy a variety of antimicrobial strategies. These include phagocytosis, in which they engulf and digest pathogens, and degranulation, in which they release a plethora of antimicrobial peptides, enzymes (e.g., lysozyme), and reactive oxygen species (ROS) to directly target and eliminate engulfed microbes. Additionally, neutrophils can undergo a specialized form of programmed cell death known as NETosis, distinct from apoptosis and necrosis, wherein they extrude chromatin fibers adorned with antimicrobial proteins into the extracellular milieu [8,9,11].

Moderate to severe COVID-19 is driven by immunopathological effects such as cytokine storms and poor adaptive immunity; robust neutrophil infiltration and dysfunction has been identified as a prominent finding in many of these more severe cases. In post-mortem studies of COVID-19 patients, neutrophil extravasation has been markedly observed in pulmonary capillaries, the myocardium, and the liver [6,13]. Furthermore, increased SARS-CoV-2 severity correlates with higher neutrophil-to-lymphocyte ratios (NLRs), which are subsequently linked to worsened clinical outcomes [14]. Neutrophils contribute to tissue damage through the release of reactive oxygen species, proteases, and neutrophil extracellular traps (NETs), which intensify the inflammatory cascade and exacerbate epithelial and endothelial damage, leading to organ failure and death in patients with ARDS [5,15]. Recent studies have documented alterations in oxidative bursts, phagocytic function, and reduced myeloperoxidase (MPO) activity in neutrophils collected from critically ill COVID-19 patients, yet these descriptive interpretations do not yet elucidate the mechanisms underlying neutrophil dysfunction and the complex interplay between virus-induced immune dyscrasias and COVID-19 disease progression [6,13,15]. Increased neutrophil recruitment and activation is similarly known to promote cytokine storms and severe disease progression in hospitalized patients with COVID-19, yet few studies have applied in vivo models to delineate the interdependence between viral infection, innate immune activation, and perturbations of neutrophil function as they contribute to clinical outcomes [16,17,18]. 

NETosis is a type of programmed cell death in neutrophils that is distinguished by the extrusion of DNA, histones, and antimicrobial proteins in the form of neutrophil extracellular traps (NETs). A key component of NETs is citrullinated histone-H3 (citH3) and neutrophil elastase (NE), both of which contribute to NETs’ antimicrobial properties [12,19]. CitH3, a post-translationally modified protein, plays a crucial role in the formation and stability of NETs, while neutrophil elastase is an enzyme that assists in the breakdown of extracellular matrix components and enhances the antimicrobial potency of NETs [20,21]. NETs constitute a double-edged sword in host defense and immunopathology during COVID-19. In the context of SARS-CoV-2, NETosis can be induced by several factors, including direct recognition of viral antigens by neutrophils, the action of pro-inflammatory cytokines like IL-8, and tumor necrosis factor-alpha (TNF-α) [6,22]. These cytokines contribute to the recruitment and activation of neutrophils, enhancing their ability to release NETs. Additionally, ROS generated as a result of respiratory bursts in neutrophils are also an important requirement for NET formation. Oxidative bursts causes post-translational modifications of nuclear and granular proteins that enable decondensed chromatin along with antimicrobial proteins to be released into the extracellular space. Moreover, their interaction with activated platelets and other immune cells in the processes of coagulation and inflammation may further activate neutrophils to undergo NETosis [17,20,23]. Initially recognized for their role in immobilizing and killing pathogens extracellularly, NETs also serve as a physical barrier that prevents viral dissemination and augments localized antimicrobial activity. However, dysregulated NET formation can contribute to tissue damage and immunopathological responses observed in severe COVID-19 cases [17,24]. Excessive NET release has been associated with endothelial dysfunction, microvascular thrombosis, and inflammation, exacerbating lung injury and systemic complications in critically ill patients. Despite an increasing amount of literature that provides insight into the clinical picture of COVID-19, much is unknown about the pathophysiology of SARS-CoV-2 as it relates to innate immunity [6,15,21]. These gaps in our understanding of the drivers of SARS-CoV-2-associated innate immune dysfunction underscore a critical need for translational studies to explore the mechanistic foundations of this disease.

The persistent emergence of SARS-CoV-2 variants emphasizes the need for adaptable and translational animal models for COVID-19 [25,26,27]. Importantly, domestic cats are both naturally and experimentally infected with SARS-CoV-2, which mimics acute COVID-19 in humans [28,29,30,31]. In the context of neutrophil biology, there are unique advantages to feline models for understanding immune responses to SARS-CoV-2. Comparative studies elucidate species-specific variations in neutrophil dysfunction and evaluate implications for disease progression. This study evaluates and characterizes neutrophil activation, the role of neutrophil extracellular traps in viral clearance, and immunopathology. Findings from our study contribute to the formulation of the global framework that characterizes the biology of neutrophils in the context of COVID-19, further translating insights from the feline viral model into clinical practice and public health. Additionally, insights provided through this study will support the development of targeted therapies that modulate neutrophil function to reduce disease severity and improve patient outcomes. Furthermore, the identification of immune pathways conserved between humans and felines should accelerate the translation of experimental findings into clinical settings, facilitating the development of more effective vaccines and therapeutic interventions against SARS-CoV-2.

## 2. Results

### 2.1. Significant Alterations in Inflammatory Markers in Plasma and Bronchoalveolar Lavage Fluid Observed in SARS-CoV-2-Infected Cats

**Figure 1:**
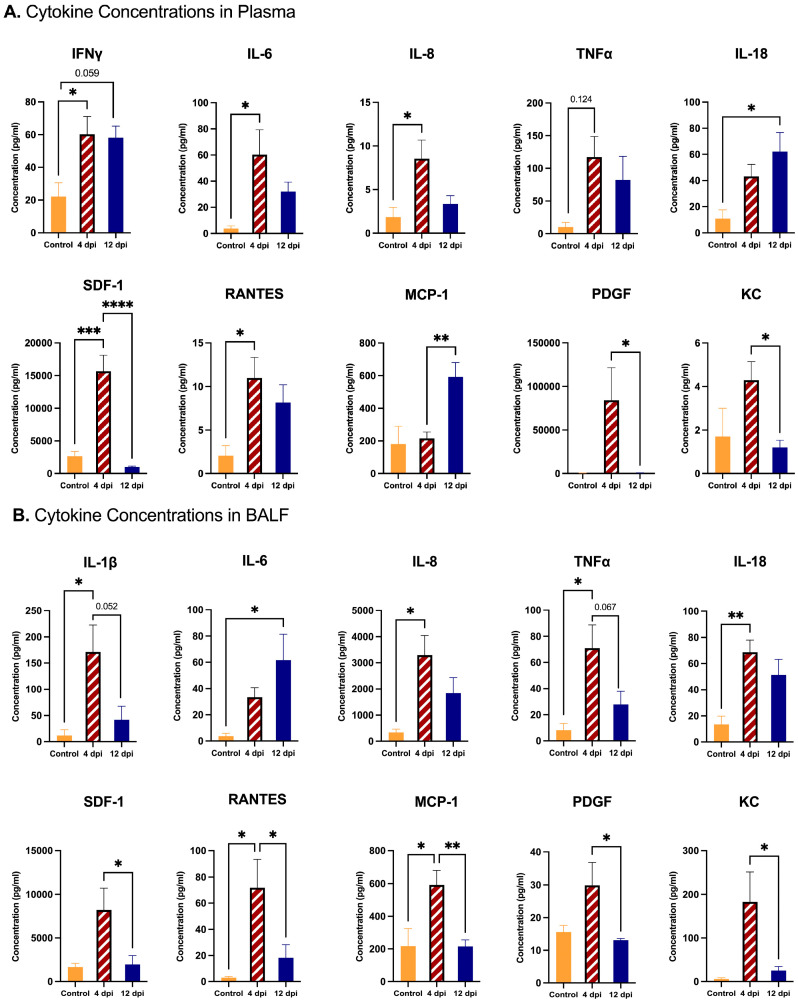
Comparison of cytokine concentrations in plasma and BALF of SARS-CoV-2-infected cats. Plasma cytokine concentrations were measured in SARS-CoV-2-infected cats 4 dpi (*n* = 12) and 12 dpi (*n* = 12) and in sham-inoculated controls (*n* = 6) using multiplex immunoassay: (**A**) IFN-γ, IL-8, TNF-α, IL-18, SDF-1, RANTES, MCP-1, PDGF, and KC show significant alterations between study groups. (**B**) Cytokine concentrations in BALF of SARS-CoV-2-infected 4 dpi cats (*n* = 12), 12 dpi cats (*n* = 12), and sham-inoculated controls (*n* = 6) were measured using multiplex immunoassay. Significant differences were observed in levels of IL-1β, IL-8, TNF-α, IL-18, SDF-1, RANTES, MCP-1, PDGF, and KC among the study groups. One-way ANOVA statistical analysis was performed. Data are represented as the mean ± SEM. * *p* < 0.05, ** *p* < 0.01, *** *p* < 0.001, **** *p* < 0.0005.

Plasma concentrations of cytokines and chemokines from a pre-selected panel were measured and compared between 4 dpi SARS-CoV-2-infected cats (*n* = 12), 12 dpi cats (*n* = 12), and sham-inoculated controls (*n* = 6) to determine differences in systemic immune responses over time using a multiplex immunoassay. The results indicated distinctive patterns of alterations between measured inflammatory markers in response to SARS-CoV-2 infection. Significantly increased levels of IFN-γ, IL-6, IL-8, SDF-1, and RANTES were observed in SARS-CoV-2-infected cats at 4 dpi compared to sham-inoculated controls. Additionally, IL-18 levels were significantly increased at 12 dpi when compared to sham-inoculated controls, underscoring a sustained inflammatory response. SDF-1, PDGF and KC levels were significantly decreased at 12 dpi relative to 4 dpi in SARS-CoV-2-infected cats. Conversely, MCP-1 was upregulated at 12 dpi compared to 4 dpi in SARS-CoV-2-infected cats, further supporting ongoing systemic inflammatory processes (*p* < 0.05, *p* < 0.01, *p* < 0.001, *p* < 0.0005) (Figure 1A). Similarly, the utilization of multiplex immunoassay for cytokine and chemokine profiling in bronchoalveolar lavage fluid (BALF) of 4 dpi SARS-CoV-2-infected cats (*n* = 12), 12 dpi cats (*n* = 12), and sham-inoculated controls (*n* = 6) revealed substantial differences in the infected cats, confirming localized lung inflammation in response to SARS-CoV-2 infection. A significant increase in levels of IL-1β, IL-8, TNF-α, IL-18, RANTES, and MCP-1 was evident in 4 dpi-infected cats compared to sham-inoculated controls. In contrast, IL-6 was significantly increased in 12 dpi infected cats when compared to sham-inoculated controls. Over the course of infection, levels of SDF-1, RANTES, MCP-1, PDGF, and KC were significantly decreased in 12 dpi cats relative to 4 dpi cats (*p* < 0.05, *p* < 0.01) (Figure 1B). A full overview of all analyzed cytokines and chemokines is available in Figure S1.

### 2.2. Increased Expression of NET-Related Markers in the Lungs of SARS-CoV-2-Infected Cats

**Figure 2:**
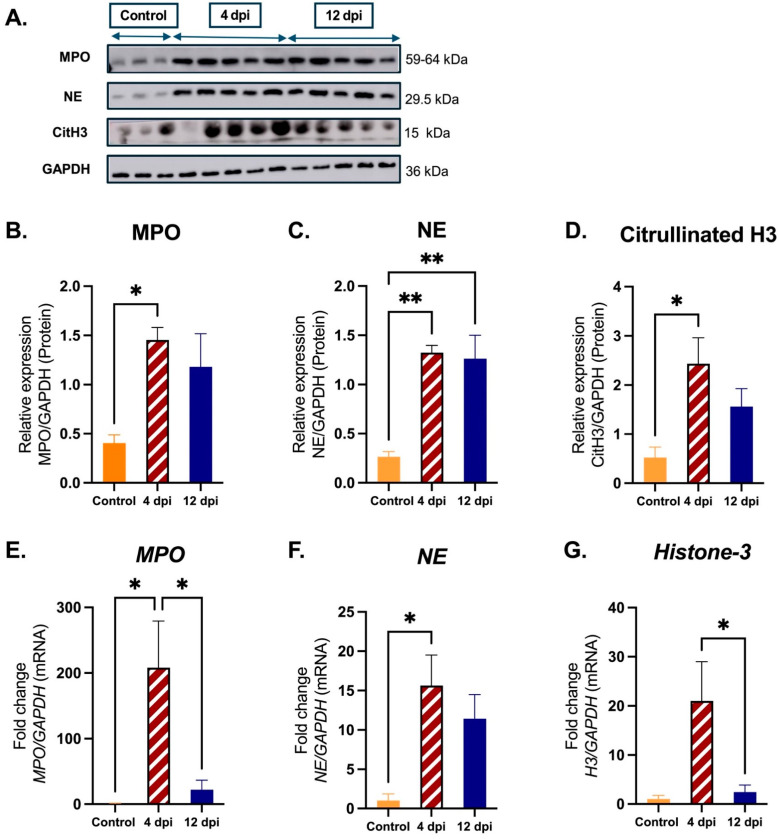
Expression of NET-related markers in lung tissues from SARS-CoV-2-infected cats. Lung tissues were collected from SARS-CoV-2-infected cats at 4 dpi (*n* = 12) and 12 dpi (*n* = 12), and from sham-inoculated controls (*n* = 6). For Western blot analyses: (**A**) samples from randomly selected SARS-CoV-2-infected 4 dpi cats (*n* = 5), SARS-CoV-2-infected 12 dpi cats (*n* = 5), and sham-inoculated controls (*n* = 3) were analyzed for protein expression of NET-related markers (MPO, NE, and citrullinated H3); objective quantification and comparisons were carried out using densitometric analysis of MPO, NE, or CitH3 protein expression relative to GAPDH: (**B**) significantly increased MPO protein was quantified in 4 dpi cats versus controls, (**C**) significantly increased NE protein was quantified in 4 and 12 dpi cats relative to controls, and (**D**) significantly increased CitH3 protein was quantified in 4 dpi cats relative to controls. For mRNA expression of NET-related markers, samples from SARS-CoV-2-infected 4 dpi cats (*n* = 12), 12 dpi cats (*n* = 12), and sham-inoculated controls (*n* = 6) were analyzed using real-time quantitative PCR analysis. Fold changes from 0 dpi were analyzed between study groups for *MPO*, *NE*, and *Histone-3*: (**E**) The fold change in mRNA expression of *MPO* relative to *GAPDH* was significantly higher in 4 dpi relative to controls and reduced 12 dpi relative to 4 dpi (**F**) The fold change in mRNA expression of *NE* relative to *GAPDH* was significantly higher in 4 dpi compared to controls. (**G**) The fold change in mRNA expression of *Histone H3* relative to *GAPDH* indicates significant reduction in expression from 4 dpi to 12 dpi. Statistical comparisons were performed using one-way ANOVA analysis. Data are represented as the mean ± SEM. * *p* < 0.05, ** *p* < 0.01.

Protein and mRNA expression levels of myeloperoxidase (MPO), neutrophil elastase (NE), and citrullinated Histone-3 (citH3)/histone-3 were quantified in SARS-CoV-2-infected cats and sham-inoculated control cats to assess differences in NETs and neutrophil activation-related markers in the lungs. Representative images show MPO, NE, and citH3 protein expression patterns across the three groups (Figure 2A). Expression of these proteins is significantly increased at 4 dpi compared to sham-inoculated controls (*p* < 0.05), with protein expression levels decreasing from 4 dpi to 12 dpi. MPO protein levels were significantly increased at 4 dpi compared to sham-inoculated controls (*p* < 0.05) (Figure 2B). NE protein levels were significantly elevated at both 4 dpi (*p* < 0.01) and 12 dpi (*p* < 0.01) relative to sham-inoculated controls (Figure 2C). citH3 protein levels were significantly increased at 4 dpi compared to sham-inoculated controls (*p* < 0.05) (Figure 2D). Quantification of mRNA from NET-related markers revealed results similar to those from Western blot analyses. *MPO* and *NE* mRNA levels significantly increased at 4 dpi compared to sham-inoculated controls (*p* < 0.05) (Figure 2E,F). *MPO* and *Histone-3* (*H3*) mRNA levels were significantly reduced at 12 dpi relative to levels at 4 dpi (*p* < 0.05) (Figure 2G). 

### 2.3. Increased Concentrations of NET-Related Markers in Plasma and Bronchoalveolar Lavage Fluid from SARS-CoV-2-Infected Cats

**Figure 3:**
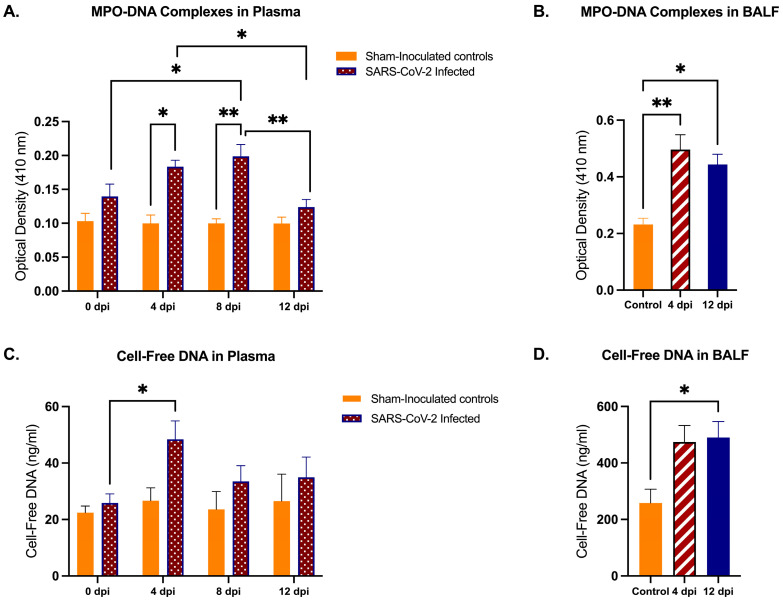
Quantification of NET-specific markers in plasma and BALF in SARS-CoV-2-infected cats. Plasma was collected from SARS-CoV-2-infected cats (*n* = 12) and sham-inoculated controls (*n* = 3) at 0 dpi, 4 dpi, 8 dpi, and 12 dpi. BALF was collected from SARS-CoV-2-infected cats at 4 dpi (*n* = 6), and 12 dpi (*n* = 12), and from sham-inoculated controls (*n* = 12). (**A**) MPO-DNA complexes in plasma were measured using MPO-DNA ELISA. Results indicate significant increases in these complexes in infected cat plasma relative to sham-inoculated controls, most markedly at 4 and 8 dpi. (**B**) MPO-DNA complexes in BALF were measured using MPO-DNA ELISA and indicate significant increases in complexes in infected cats relative to controls at both 4 and 12 dpi. (**C**) Cell-free DNA in plasma measured using Quanti-pico dsDNA assay showed a significant increase in infected cats from inoculation to 4 dpi. (**D**) Cell-free DNA in BALF measured using Quanti-pico dsDNA assay showed significant increase in DNA detection at 12 dpi relative to control cats. Statistical comparisons were performed using two-way ANOVA analysis and one-way ANOVA analysis. Data are represented as the mean ± SEM * *p* < 0.05, ** *p* < 0.01.

NET-related markers, namely, MPO-DNA complexes and cell-free DNA, were quantified in the plasma and BALF of SARS-CoV-2-infected cats using MPO-DNA ELISA and quanti-pico dsDNA assays, respectively. MPO-DNA complexes were significantly increased in the plasma of infected cats at 4 dpi (*p* < 0.05) and 8 dpi (*p* < 0.01) as compared to controls; there was significant reduction by 12 dpi versus 4 dpi (*p* < 0.05) and 8 dpi (*p* < 0.01) (Figure 3A). Similarly, MPO-DNA levels in BALF were increased in infected cats at both 4 dpi (*p* < 0.01) and 12 dpi (*p* < 0.05) relative to controls (Figure 3B). Cell-free DNA in plasma showed similar trends, with significant increases at 4 dpi compared to 0 dpi (*p* < 0.05) in SARS-CoV-2-infected cats (Figure 3C). However, in BALF, cell-free DNA levels were significantly increased at 12 dpi versus 0 dpi (*p* < 0.05) but increases lacked significance at 4 dpi compared to sham-inoculated cats (Figure 3D). 

### 2.4. NET-Specific Markers Are Localized with Infiltrated Neutrophils in Lung Tissues of SARS-CoV-2-Infected Cats

**Figure 4:**
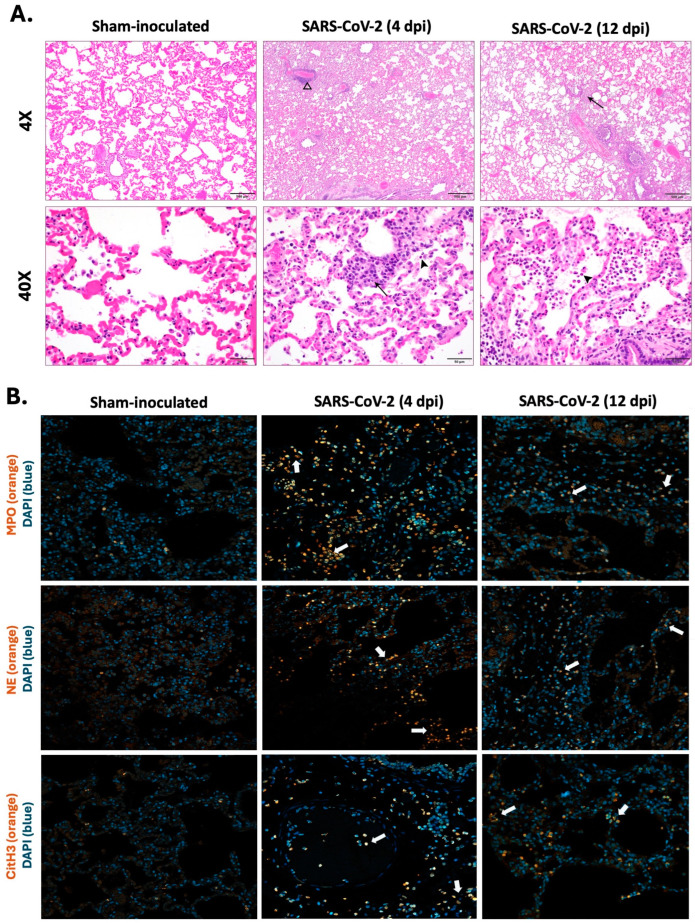
Visualization of neutrophil infiltration with NET-related markers in lung tissues of SARS-CoV-2-infected cats. Histopathological analysis and immunofluorescence staining were performed on lung tissues collected from SARS-CoV-2-infected cats at 4 dpi (*n* = 12) and 12 dpi (*n* = 12) and sham-inoculated controls (*n* = 6). (**A**) Representative images of histopathological lesions in infected cats; 4 dpi cats showed increased perivascular infiltration of inflammatory cells (open arrowhead) including neutrophils (arrow/arrowhead), while 12 dpi cats showed an intense neutrophil infiltration (arrow/arrowhead). (**B**) Representative images for MPO, NE, and citrullinated H3 in the lungs of SARS-CoV-2-infected 4 dpi cats, SARS-CoV-2-infected 12 dpi cats, and sham-inoculated controls. NETs were identified by co-localization of DNA (blue) with MPO/NE/citrullinated H3 (orange). White arrows show activated neutrophils with an indication of NET formation and release within infected lungs of 4 dpi and 12 dpi cats. Magnification: (**A**) 4x, scale bar = 500 µm; 40×, scale bar = 50 µm; (**B**) 20x, scale bar = 50 µm.

Lungs were collected at 4 dpi and 12 dpi and compared to sham-inoculated controls. In sham-inoculated controls, histopathological analysis revealed largely preserved alveolar architecture with minimal inflammation. In contrast, SARS-CoV-2-infected lungs exhibited progressive pathology. On day 4, there was perivascular infiltration of inflammatory cells (Figure 4A), whereas on day 12 there was intense neutrophil infiltration. Notably, the infected tissues showed increased intra-alveolar neutrophils admixed with edema and fibrin. The neutrophils extended to interstitium as well as to bronchioles (Figure 4A).

Immunofluorescence assay (IFA) staining highlighted the presence of NET-related markers with infiltrated neutrophils, suggesting active NET formation during infection. There are marked increases in MPO, NE, and citH3 at 4 dpi compared to 12 dpi, suggesting more localized and intense neutrophil activation at 4 dpi with a notable reduction in extent and intensity of the markers at 12 dpi (Figure 4B). 

### 2.5. A Positive Correlation Is Evident between Histopathological/Clinical Scores and Neutrophil Activation/NET-Related Markers during SARS-CoV-2 Infection in Cats

**Figure 5:**
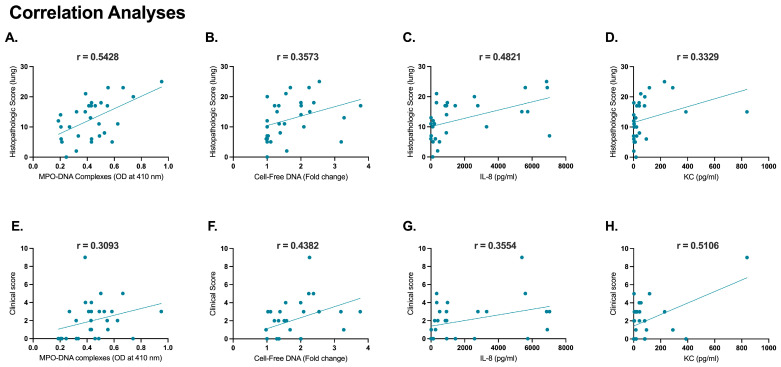
Correlation analyses of histopathology scores and clinical scores with neutrophil activation and NET-related markers. Positive correlations were evident following the correlation analysis conducted for histopathological scores from right cranial lung lobes from SARS-CoV-2-infected 4 dpi cats (*n* = 12), 12 dpi cats (*n* = 12), and sham-inoculated controls (*n* = 6) with NET-specific markers: (**A**) MPO-DNA complexes (r = 0.5428, *p* = 0.001), (**B**) cell-free DNA (r = 0.3573, *p* = 0.0526); and neutrophil activation associated markers: (**C**) IL-8 (r = 0.4831, *p* = 0.0069) and (**D**) KC (r = 0.3329, *p* = 0.0723). Positive correlations were evident following the correlation analysis conducted for the clinical scores of these cats with NET-specific markers revealed positive correlations: (**E**) MPO-DNA complexes (r = 0.3093, *p* = 0.0962), (**F**) cell-free DNA (r = 0.4382, *p* = 0.01); and neutrophil activation-associated markers: (**G**) IL-8 (r = 0.3554, *p* = 0.0539) and (**H**) KC (r = 0.5106, *p* = 0.0039). Statistical comparisons were performed using Pearson coefficient correlation analysis. Data are represented using the Pearson correlation coefficient (r).

Correlation analyses present any correlation between histopathological scores and clinical scores as disease severity parameters versus NET-specific (MPO-DNA complexes and cell-free DNA of BALF) and neutrophil activation-related markers (IL-8, KC of BALF) to determine the strength and direction of the association. Parameters were obtained from SARS-CoV-2-infected 4 dpi cats (*n* = 12), 12 dpi cats (*n* = 12), and sham-inoculated controls (*n* = 6). Collectively, all parameters showed varying degrees of positive correlation between the two disease severity variables, as indicated by the Pearson correlation coefficient. MPO-DNA complexes (r = 0.5428, *p* = 0.001), cell-free DNA (r = 0.3573, *p* = 0.0526), and IL-8 (r = 0.4831, *p* = 0.0069) show a moderately strong positive correlation with histopathological scores, while KC demonstrate a weaker positive correlation (r = 0.3329, *p* = 0.0723) (Figure 5A–D). Cell-free DNA (r = 0.4382, *p* = 0.01) and KC (r = 0.5106, *p* = 0.0039) show a significant, moderately positive correlation with clinical scores while MPO-DNA ELISA (r = 0.3093, *p* = 0.0962) and IL-8 (r = 0.3554, *p* = 0.0539) show a relatively weaker positive correlation **(**Figure 5E–H). 

### 2.6. RNA Sequencing Data Reveal a Significant Association between SARS-CoV-2 Infection, Innate Immune Response, and Neutrophil Activation in Cats

**Figure 6:**
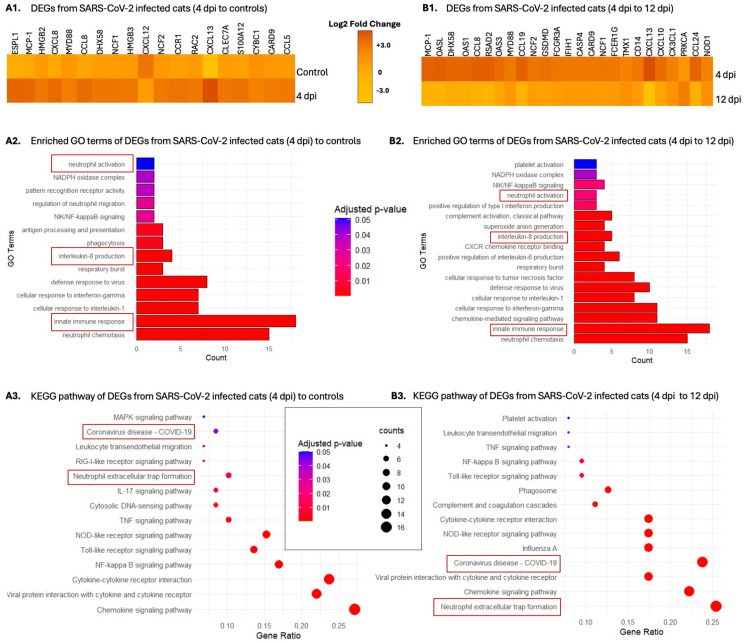
Heatmap and enrichment analysis of differentially expressed genes in SARS-CoV-2-infected cats. The figure includes heatmaps and bar plots illustrating the enriched gene ontology (GO) terms and Kyoto Encyclopedia of Genes and Genomes (KEGG) pathways associated with differentially expressed genes in SARS-CoV-2-infected cats. (**A1**,**B1**) Heatmaps showing the expression levels of differentially expressed genes across various conditions and time points revealed significant increases of neutrophil activation-related genes at 4 dpi compared to both sham-inoculated controls and 12 dpi SARS-CoV-2-infected cats. (**A2**,**B2**) Bar plots of enriched GO terms suggest significant upregulation of neutrophil activation and recruitment pathways with an overall increase of innate immune response in 4 dpi cats compared to both sham-inoculated controls and 12 dpi SARS-CoV-2-infected cats. (**A3**,**B3**) Bubble plot of top enriched KEGG pathways supporting the association of DEGs from 4 dpi cats with COVID-19 and neutrophil trap formation compared to DEGs from sham-inoculated controls and 12 dpi SARS-CoV-2-infected cats.

Comprehensive gene expression analysis was conducted using bulk RNA sequences from the lungs of infected cats to investigate the differentially expressed genes (DEGs) and corresponding biological processes and pathways affected by SARS-CoV-2 infection, with an emphasis on innate immune responses. Comparisons between SARS-CoV-2-infected 4 dpi cats and sham-inoculated controls reveal a total of 3544 DEGs which are upregulated and 1708 genes that are downregulated (Figure S2A). Significant upregulation of genes related to innate immune responses and neutrophil activation was noted, including ESPL1, MCP-1, HMGB2, CXCL8, MYD88, CCL8, DHX58, NCF1, HMGB3, NCF2, CCR1, RAC2, CXCL13, CLEC7A, S100A12, CYBC1, CARD9, and CCL5 (Figure 6(A1)). Selected genes were further analyzed using GO and KEGG pathway analysis. GO analysis identified genes involved in several neutrophil and NETosis pathways, including innate immune responses, neutrophil activation, and IL-8 production (*p* < 0.05) (Figure 6(A2)). Notably, KEGG pathway analyses from these DEGs highlight their function in COVID-19 and neutrophil extracellular trap formation (Figure 6(A3)). 

DEG analyses between SARS-CoV-2-infected 12 dpi cats and sham-inoculated controls identified 860 downregulated genes and 2904 upregulated genes (Figure S2B). There was a significant decrease in neutrophil activation and innate immune response-related genes including LTF, CXCL8, CAMP, ELANE, ADAM8, and OAS1 (Figure S3). Emphasis on the innate immune responses and related pathways revealed that although the pathways seem related, they are not significantly related, but they loosely support reduction in neutrophil involvement and inflammation during the course of infection (Figure S3). 

DEG analysis between 4 dpi cats and 12 dpi cats revealed 3188 downregulated genes and 4776 upregulated genes (Figure S2C). Significant increases in inflammation and innate immune response-related pathways were noted, including MCP-1, OASL, DHX58, OAS1, CCL8, RSAD2, OAS3, MYD88, NCF2, GSDMD, FCGR3A, IFIH1, CASP4, CARD9, NCF1, FCER1G, TMX1, CD14, CXCL13, CXCL10, CX3CL1, PRKCA, CCL24, and NOD1 (Figure 6(B1)). The enrichment analysis revealed that the DEGs were involved in numerous innate immune pathways, including the innate immune response, neutrophil activation, and IL-8 production, suggesting the modulation of pathways is more intense during the early acute phase of infection (Figure 6(B2)). KEGG pathway analysis further revealed the association of the selected genes with COVID-19 and NET formation at 4 dpi compared to 12 dpi (Figure 6(B3)).

## 3. Discussion

In this study, we investigated neutrophil kinetics and related immune responses in the context of COVID-19 in a feline model, which has been shown to mimic acute COVID-19 in hospitalized patients [30,31], underscoring this animal model as translational for the study of disease in people. Enhanced production of inflammatory cytokines and chemokines during SARS-CoV-2 infection orchestrates a robust immune response, pivotal in modulating and mitigating disease progression [32,33,34,35]. This study delineates a clear temporal pattern in the cytokine response to SARS-CoV-2 infection with an initial surge in pro-inflammatory cytokines including IFN-γ, IL-6, and TNF-α at 4 dpi, indicating immediate immune response and a decline at 12 dpi, reflecting a regulatory mechanism to alleviate the immune response and avoid excessive tissue damage. These results are consistent with what has been observed in people and other in vivo models of SARS-CoV-2 infection [36,37,38]. Interestingly, a noticeable shift in cytokine profiles in plasma from SARS-CoV-2-infected cats at 12 dpi shows marked increases in IL-18 and MCP-1, further underscoring persistent inflammation and a systemic inflammatory condition that could impact other organs such as the liver, kidneys, intestines, brain, and heart [39,40,41]. Observed modulations of cytokine and chemokine levels in BALF mirror localized responses within the lungs, the primary site of infection. Elevated levels in BALF in infected cats not only corroborate the presence of intense inflammation but align with findings in human COVID-19. The sustained increase in IL-6 level measured in BALF through 12 dpi supports the continuous engagement of IL-6 in immune mechanisms, likely to address persistent viral particles and tissue repair, which may contribute to pathology seen in severe cases. This finding supports that the conclusion that sustained IL-6 expression is linked with prolonged disease severity [42]. 

Notably, the majority of these cytokines are directly associated with the recruitment and activation of neutrophils, which are pivotal in the initial immune response but also contribute to inflammatory pathology if excessively activated [43]. Elevations in IL-8, SDF-1, KC, and RANTES highlight a significant role in promoting neutrophil activation and NETosis which could, in part, explain prolonged inflammation observed in infected cats. Persistently elevated IL-8 and IL-18, particularly at 4 dpi, suggest sustained recruitment and activation of neutrophils. The modulation of SDF-1, MCP-1, and KC levels also supports neutrophil recruitment, influencing the extent and duration of NET formation [36,37,42]. Moreover, the upregulation of RANTES suggests platelet activation and thrombosis, in which NETs play a pivotal role [44]. Overall, characterization of NETosis is crucial for delineating the pathogenesis of SARS-CoV-2 infections. This comprehensive analysis demonstrates dynamic changes in cytokine and chemokine profiles in SARS-CoV-2-infected cats, offering insights into systemic and localized immune responses. Importantly, the observed cytokines and chemokines play a functional role in neutrophil activation and NETosis with IL-8 and IL-18 identified as critical activators [16,20]. The modulation of SDF-1, MCP-1, and KC levels further suggests their role in neutrophil recruitment into infection sites, underscoring their potential impact on inflammation and tissue damage during SARS-CoV-2 infection [16,20,45].

A hallmark of COVID-19 in hospitalized ICU patients is the excessive production of NETs in their lungs, leading to detrimental outcomes [6]. Hence, understanding the involvement of NETosis in a feline model for COVID-19 is an important translational step. NETs consist of several components that are deliberately excreted from activated neutrophils, such as extracellular DNA strands and associated MPO, NE, and citrullinated histone-3 [17]. Data reveal a marked increase in the protein expression of NET-related markers such as MPO, NE, and citH3 at 4 dpi, indicating a strong activation of neutrophils and the formation of NETs early in the infection process. The significant increase in the expression of citH3, a specific marker of NETosis, at 4 dpi highlights likely NET formation via several pathways [46,47]. Cell-free DNA and citH3 are not produced in the neutrophil cell death pathways, which are independent from NETosis [48,49]. The decrease in these protein markers at 12 dpi supports a potential regulatory mechanism to prevent excessive tissue damage. The mRNA levels for MPO and NE did not show a significant increase, suggesting post-transcriptional regulation or rapid protein turnover as potential factors influencing NETosis pathways. This discrepancy between mRNA and protein levels highlights the complex regulation of neutrophil responses at multiple levels of gene expression and protein synthesis [22,50].

Elevation of NET-related markers during systemic infection indicate NETosis and neutrophil activation as a part of the innate immune response to the initial viral infection. Observed trends that peak at 4 dpi followed by a gradual decline in plasma suggest the NETosis process is most aggressive during the early, acute phase of infection. Increases in NET-specific markers in both plasma and BALF, namely MPO-DNA complexes and cell-free DNA, suggest neutrophil activation and NETosis occur in response to SARS-CoV-2 infection both locally and systemically. Given significant increases observed in BALF, it is plausible that initial neutrophil activation is predominantly localized in the lungs where direct contact with SARS-CoV-2 virus occurs. A lung environment, enriched with virus and inflammatory cytokines, should serve as an ideal site for the activation of neutrophils. However, the additional presence of elevated NET-related markers in plasma suggest that systemic activation occurs as well, prompted by circulating inflammatory cytokines such as IL-8 and IL-18 [5,51]. Circulation of NETs may activate neutrophils to translocate from the lungs back to the bloodstream as well. A recirculation such as this may explain sustained levels of NET markers in plasma over time. Moreover, activated neutrophils could migrate back into the bloodstream upon concluding their functions in the lungs, a crucial step in amplifying systemic immune responses. Systemic activation further suggests an attempt by the host response to mobilize innate defenders and prepare other potential sites to combat infection [5,47]. Overall, results indicate a marked increase in neutrophil activation and NETosis-related markers in the infected lung tissues at 4 dpi with a subsequent decline by 12 dpi, suggesting a significant role of neutrophil responses during the course of SARS-CoV-2 infection in cats. 

Colocalization of NET-specific markers with neutrophil recruitment to the lung is well evidenced in hospitalized COVID-19 patients and results in more severe outcomes such as impaired gas exchange, dyspnea, acute lung injury, and acute respiratory distress syndrome (ARDS) [52]. Histological and immunofluorescence analyses provide visual evidence of pathology and hyperactivated innate immune responses in lung tissues from SARS-CoV-2-infected cats. Histopathological analysis and scoring provide further insight into the relationship between neutrophil activation and pulmonary tissue damage. Increased cellular infiltration and alterations in lung architecture of infected cats indicate progressive inflammation through varied stages of acute infection. Similar to people with COVID-19, NETs are predominantly observed within alveolar spaces and around small airways [53]. Increased intensity of NET formation, as indicated by immunofluorescence assay, correlates with areas of amplified neutrophil infiltration and suppurative inflammation similar to severe COVID-19 cases in which excessive NET formation is noted to contribute to alveolar damage. The presence of NETs with increased cellular infiltration and associated pulmonary damage support that NETs are both a vital part of the robust host immune response aiming to contain virus but also contributing to pathology [53,54]. Reduction in NETs by 12 dpi support resolution of initial immune responses and depletion of neutrophil reserves. This progression and resolution of NETosis likely have varied timelines and dynamics, influenced by differences in immune system functions and clinical management.

Correlation analyses aim to elucidate the association between disease severity parameters and innate immune response, particularly focusing on the role of neutrophils and NETs in the pathogenesis of the disease. Moderately strong correlations suggest that if one variable increases the other would increase as well at a relatively consistent rate. A weaker positive correlation suggests that the variables are related, yet the impact on each other’s changes is less consistently associated with changes in others. Hence, the experimented parameters certainly correlate well with tissue damage while the clinical progression of the disease may be influenced by many other variables. A strong correlation of NET markers with histopathological scores correlates the formation of NETs with exacerbation of tissue injury during SARS-CoV-2 infection [54]. The weaker correlation of MPO-DNA complexes and IL-8 with clinical scores suggests that while these parameters are linked to the clinical scores, the relationship may be more complex and multi-factorial.

Bulk RNA sequencing provides valuable insight into molecular mechanisms underlying SARS-CoV-2 infection in cats, with a specific focus on neutrophil activation and related innate immune response and functions. Our findings reveal significant upregulation of genes associated with innate immunity and neutrophil activation in 4 dpi SARS-CoV-2-infected cats, highlighting the involvement of neutrophils in host responses in addition to viral evasion in the early acute phase. Notably, the enrichment analyses of DEGs identified key biological processes and pathways involved in neutrophil activation and NET formation, emphasizing their role in the pathogenesis of COVID-19 in the feline model. Conversely, a notable decrease in the expression of neutrophil activation-related genes at 12 dpi suggests potential dampening of the inflammatory response in late stages of infection. This temporal modulation of neutrophil pathways underscores the dynamic nature of the host response to SARS-CoV-2, with distinct patterns of gene expression observed during the early acute phase versus late acute stages of infection.

Moreover, the presence of cell-free DNA in plasma and BALF at 12 dpi further suggests incomplete clearance and may thus promote chronic inflammation by acting as damage-associated molecular patterns [51,55,56]. In the context of vital NETosis, it should mirror apoptosis following viral clearance, in which no intracellular components are released externally. The presence of cell-free DNA at high levels suggests the incidence of suicidal NETosis [57,58,59]. This form of NETosis involves the extensive release of NETs, which contain DNA strands, histones, and proteases. These components, particularly histones, are cytotoxic and can severely damage endothelial and epithelial cells. Such damage not only increases vascular permeability but also compromises the integrity of epithelial barriers, facilitating further pathogen invasion and fluid leakage into tissues like the lungs [55,60,61,62,63]. Additionally, the extracellular components from NETs, such as cell-free DNA and histones, act as pro-inflammatory signals that can attract further immune cells, leading to a hyper-inflammatory state [47,51,64,65].

From a therapeutic standpoint, our data suggest that therapeutic strategies targeting NETs could be beneficial, particularly if implemented in the early phase of infection when NET formation is most aggressive. In addition, the correlations of NET-related markers, including IL-8, KC, dsDNA, and MPO-DNA complexes, with histopathological scores and clinical scores further imply their capacity to act as therapeutic targets. Therapeutic interventions blocking excessive NETosis or stimulating its resolution may avoid tissue damage and reduce disease severity. Several studies have focused on the alteration of and inhibition of NETs to mitigate associated pathology during SARS-CoV-2 infection [56,58,66]. Research includes the use of DNases, inhibition of PAD-4, and reduction of IL-8, which is proven to reduce NET production which has effectively reduced lung inflammation and damage during infection [2,58,67,68,69,70,71]. However, the timing of such interventions is crucial, as the suppression of NETs might also interfere with their protective functions against viral spread. Therefore, this necessitates a delicate balance between the beneficial and detrimental roles of NETs.

The translation of findings from a feline model to human clinical applications holds significant promise for advancing medical treatments, especially concerning interventions targeting NETs in SARS-CoV-2 infections [25,26,27,72]. Feline models, which closely mimic human responses to SARS-CoV-2, provide essential insights into the pathophysiology of the disease and the effectiveness of potential therapies [27,30,31,73]. This research is instrumental in establishing the optimal dosing, timing, and delivery methods that can later be adapted for human clinical trials. For instance, evidence from feline studies demonstrating the benefits of reducing NET formation can directly inform the development of similar therapeutic strategies in humans, aiming to alleviate severe symptoms and reduce mortality associated with COVID-19. Furthermore, the controlled environment of feline studies offers a clearer understanding of the therapeutic mechanisms, enhancing the predictability and translatability of outcomes to human settings. The insights gained from such studies are crucial for designing human trials that are more targeted and efficient, potentially accelerating the regulatory approval process and the availability of new treatments. 

For the first time in feline SARS-CoV-2 research, we show that excessive neutrophil recruitment at the infection site is functionally active and that these activated neutrophils undergo NETosis. Further, NET-specific markers coincide with histopathological damage in the lungs. By demonstrating a similar correlation to those observed in human studies as well as other animal studies [5,60], our findings validate the feline model as a relevant and reliable translational model to study innate immune responses during COVID-19. Nevertheless, additional research is required to definitively determine the impact of various NETosis pathways on tissue damage and disease progression. Although our study provides valuable insights, it does not comprehensively address other confounding factors that may affect tissue damage during SARS-CoV-2 infection. Such factors could encompass the roles of different immune cell types, the effects of concurrent bacterial infections, or the influence of pre-existing conditions on the severity of the disease. The sample size, although adequate for statistical analysis, limits the generalizability of the results. Also, while specific for NETosis and neutrophil activation, selected markers may be regulated via other mechanisms and pathways that were not evaluated during this study. Addressing these gaps will be essential for the development of targeted therapies that can modulate NET formation without undermining their fundamental defensive functions. 

In conclusion, neutrophils remain a dichotomy in our host immune responses. Significant upregulation of NETosis in acute SARS-CoV-2 infections highlights a sometimes excessive, dysfunctional response of neutrophils as host defense against viral infection. Subsequent reduction in these markers for NETosis suggests a sophisticated regulatory mechanism that modulates intensity of inflammation and protects from sustained tissue damage. Better understanding of the peaks and reductions of these responses will highlight their contribution as effective targets for therapeutic interventions aimed at modulating neutrophil activation and inflammation to mitigate disease severity. Furthermore, continued validation of the feline model for COVID-19 expands the repertoire of animal models available for preclinical research, facilitating the development and evaluation of these novel therapeutic approaches.

## 4. Materials and Methods

### 4.1. Animals and Ethics Approval

A total of thirty specific pathogen-free (SPF) cats were obtained from Marshall Bioresources (North Rose, NY, USA) and acclimatized for a duration of 30 days in accordance with standard guidelines as previously published [31]. Briefly, animals intended for SARS-CoV-2 infection (*n* = 24) were housed at Animal Biosafety Level 3 (ABSL-3) facilities, whereas animals intended for sham inoculation (*n* = 6) were housed at standard animal facility at Oklahoma State University. All cats had access to water and food ad libitum. Cats were assessed for health parameters including baseline body weight, body temperature, and clinical examination prior to inoculation to ensure their overall health. The study was approved by the ethics committee at OSU on the use of animals (IACUC 20-48 STW). 

### 4.2. Virus Propagation and SARS-CoV-2 Infection

A human isolate of the SARS-CoV-2 virus B.6.617.2 (Delta variant) was obtained from BEI Resources (Manassas, VA, USA) and propagated as previously published [31]. In brief, the virus was passaged seven times on Vero cells (CCL-81; ATCC, Manassas, VA, USA) in Dulbecco’s Modified Eagle Medium (DMEM, Gibco, Carlsbad, CA, USA) consisting of 5% fetal bovine serum (Hyclone, Logan, UT, USA) in addition to 1% Penicillin-Streptomycin (Gibco, Carlsbad, CA, USA). The obtained viral stocks were titrated based on TCID_50_ using the Reed and Muench calculation [74] as previously described [30]. 

**Figure 7:**
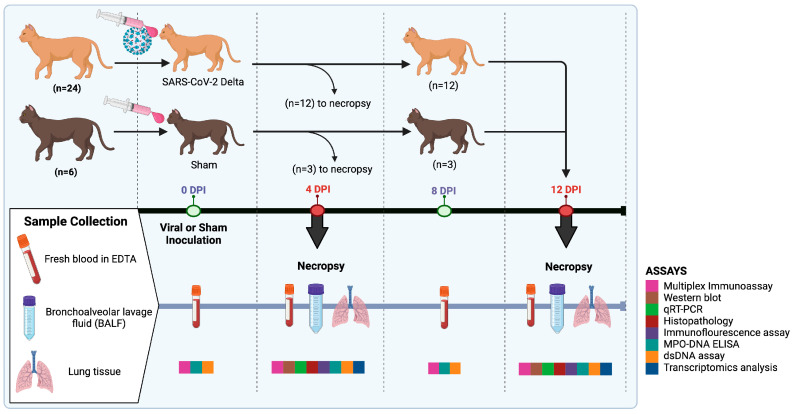
Experimental design. This diagram illustrates the experimental timeline and procedures throughout the study. The study consisted of a total of 30 cats, 24 of which were inoculated with SARS-CoV-2 (Delta variant), and 6 of which were sham-inoculated. Twelve SARS-CoV-2-infected and 3 sham-inoculated were euthanized and samples collected 4 DPI. The remaining twelve infected and 3 sham-inoculated cats were euthanized for sample collection 12 DPI. Blood, tissues, and bronchoalveolar lavage were all collected for analysis. Image made in Biorender.

Age-matched (1-year-old) and sex-matched (15 male and 15 female) cats were randomly assigned to study groups as previously published [31]. Prior to inoculation procedures, all cats were sedated via intramuscular administration of ketamine (4 mg/kg), dexmedetomidine (20 µg/kg), and butorphanol (0.4 mg/kg) as previously published and described [30,31]. Concisely, a total of twenty-four cats were intratracheally and/or intranasally inoculated with SARS-CoV-2 (Delta) virus, while 6 of the cats were inoculated with DMEM as sham-inoculated controls. Cats (*n* = 24) were inoculated with an equal dose of virus, 1.26 × 10^6^ TCID_50_ in a weight-dependent manner using 1 mL of DMEM. The remaining controls (*n* = 6) were inoculated with 1 mL DMEM. Of the thirty cats, fifteen were sedated and humanely euthanized using pentobarbital >80 mg/kg at 4 days post-infection (dpi) and the rest of the cats at 12 dpi. Each time point included SARS-CoV-2-infected cats (*n* = 12) and sham-inoculated cats (*n* = 3). Following euthanasia, necropsy occurred for tissue collection (Figure 7).

### 4.3. Clinical Evaluation

Clinical evaluation was carried out by a licensed veterinary practitioner utilizing a modified clinical scoring system for each considered factor (0 = normal, 1 = mild, 2 = moderate, 3 = severe) as previously published and stated [30,31]. In brief, all animals were monitored twice daily at minimum throughout the duration of the study, and clinical data were documented for each time point. The clinical parameters were then summated to gain an overall clinical score per animal and each time point.

### 4.4. Sample Collection and Processing

A total volume of 4–5 mL of whole blood was collected in EDTA from all cats by jugular puncture, following sedation at 0, 4, 8, and 12 dpi (Figure 7). Plasma was immediately separated from blood by centrifugation at 2000× *g* for 10 min at 4 °C and plasma was stored at −80 °C till further analysis [75]. BALF was collected from all cats in both time points (4 dpi and 12 dpi), post euthanasia (Figure 7). Briefly, a sterile endotracheal tube was placed in the trachea and 80 mL of sterile phosphate-buffered saline (PBS, Gibco, Carlsbad, CA, USA) was instilled into the lungs through the tube. The saline solution was allowed to settle, and the fluid was then gently aspirated back into a conical tube [76]. Supernatants were collected by centrifugation of the content at 400× *g* for 7 min at 4 °C [77]. Supernatants were transferred into new Eppendorf tubes and stored at −80 °C till further analysis. Equal volumes of BALF were collected in all cats. Right cranial lung tissues were collected from all the cats at necropsy on both 4 dpi and 12 dpi (Figure 7). Tissues were subdivided and either frozen at −80 °C or collected into tissue cassettes and fixed for 5 days in 10% neutral-buffered formalin before transferring to ethanol for 3 days as previously published [31]. Viral quantification and analyses of all animals were performed via droplet digital PCR as detailed in Tamilselvan et al. Results confirmed the presence of viral loads in all infected cats, while no infection was detected in sham-inoculated controls [31].

### 4.5. Multiplex Immunoassay for Cytokine Profiling

Plasma and BALF samples were utilized to measure systemic and respiratory cytokine concentrations respectively, using a commercially available feline cytokine–chemokine magnetic bead panel ELISA kit (Millipore-Sigma, Burlington, MA, USA) according to the manufacturer’s instructions. A comprehensive set of soluble cytokine molecules (s-Fas, TNFα, IL-12p40, SCF, PDGF-BB, IL-13, IL-18. IL-6, IL4, IL-2, GM-CSF, KC, RANTES, SDF-1, FLT-3L, IL-1β, IFNγ, MCP-1, and IL-8 were detected using a composite panel of microspheres coupled with capture antibodies [78]. In summary, 25 μL of plasma or BALF was combined with 25 μL of antibody-immobilized beads and 25 μL of assay buffer or matrix in a 96-well plate and incubated overnight at 4 °C on a shaker along with the standards. Subsequently, 25 μL of biotinylated detection antibodies was added to each well following the addition of streptavidin-phycoerythrin. The fluorescence was measured by the Bio-Plex^®^ 200 multiplex detection system (Bio-Rad, Hercules, CA, USA). Data were acquired and processed using the Bio-Plex manager software (v 6.0.) (Bio-Rad, Hercules, CA, USA).

### 4.6. RNA and Protein Isolation from Lung Tissues

Total RNA and proteins were isolated from the cranial lungs of all cats using the TRI-reagent method (MRC, Cincinnati, OH, USA) according to the manufacturer’s instructions. Briefly, 50–100 mg of lung from each cat was homogenized in TRI reagent and separated into phases using 1–bromo–3–chloropropane (BCP MRC, Cincinnati, OH, USA). RNA, and proteins were isolated from the aqueous phase and organic phase, respectively. RNA was precipitated using isopropanol and then purified using ethanol washes. Air-dried pellets were resuspended in nuclease-free water and stored at −80 °C until further analysis. Proteins were precipitated using acetone and then purified using guanidine hydrochloride, ethanol, and glycerol washes. The pellets were resuspended in 1% sodium dodecyl sulfate (SDS) and stored at −20 °C for further analysis [79,80].

### 4.7. Western Blot

The total proteins from lung of all cats were quantified using the dye reagent concentrate according to the manufacturer’s recommendations (Bio-Rad, Hercules, CA, USA). Protein concentrations were obtained using bovine serum albumin as the reference standard (Bio-Rad, Hercules, CA, USA). The readings were acquired using the spectrometric reader at 595 nm absorbance. Quantified protein samples were processed in 1X SDS sample buffer containing 0.06 M Tris (pH 6.8), 2.1% (*w*/*v*) SDS, 5% (*v*/*v*) glycerol, and 1% (*v*/*v*) 2-mercapto-ethanol. Equal amounts of the samples (20 μg) were separated on 10% SDS PAGE gels along with a pre-stained protein ladder (ThermoFisher Scientific, Waltham, MA, USA). Following SDS-PAGE, proteins were transferred onto a nitrocellulose membrane in a semi-dry electro-blotting apparatus (Transblot, Biorad, Hercules, CA, USA). Membranes were blocked for 1 h at room temperature with 5% skim milk before immunodetection. The membranes were then incubated overnight at 4 °C with intermittent shaking in respective primary antibodies: Myeloperoxidase in 1:500 dilution (MPO, FabGennix, TX, USA), Neutrophil elastase in 1:500 dilution (NE: Invitrogen, Thermofisher, Wilmington, DE, USA), citrullinated histone H3 in 1:1000 dilution (Cit.H3, Abbomax, CA, USA), and housekeeping protein Glyceraldehyde 3-phosphate dehydrogenase in 1:2000 dilution (GAPDH, (Abclonals, MA, USA) as the loading control. Membranes were incubated with a horseradish peroxidase-conjugated goat anti-rabbit secondary antibody in dilution 1:2000 (ThermoFisher Scientific, Waltham, MA, USA) for 1 h at room temperature. The signal was developed by adding chemiluminescent peroxidase substrate (ThermoFisher Scientific, Waltham, MA, USA) and images were acquired with Amersham Imager 600 (GE Healthcare, Pittsburg, PA). The protein band intensities were quantified using Image J software (v 1.8.0) and the relative expression of the target proteins was normalized to the reference protein, GAPDH during interpretation.

### 4.8. Quantitative Real-Time PCR

The purity of the total RNA from the lung was evaluated and quantified spectrophotometrically using NanoDrop ND-1000 (Thermo Fisher Scientific, Wilmington, DE, USA). RNA from all cats were diluted to equal amounts (1000 ng) and reverse transcribed to cDNA using superscript II reverse transcriptase according to the manufacturer’s recommendations (Invitrogen, Carlsbad, CA, USA) in individual reactions. The PCR reactions were run using SYBR green PCR master mix (Thermo Fisher Scientific, Wilmington, DE, USA). Primers were designed using the IDT-DNA website as listed in Table S1. The final reaction mixture (20 μL) consisted of 10 μL 2X SYBR green PCR master mix, 1 μL of the 10 μM forward and reverse primers of the gene of interest (MPO, NE, and Histone 3), 5 μL of the cDNA template with 4 μL nuclease-free water which was then added to a 96 well plate with nuclease-free water as the negative control. The qRT-PCR was performed and analyzed using QuantStudio 6 Pro Real-Time PCR Systems (Applied Biosystems, Carlsbad, CA, USA) according to the manufacturer’s instructions. The thermal cycle was as follows; initial denaturation step at 95 °C for 10 min, followed by 40 cycles at 95 °C for 15 s, 60 °C for 60 s, followed by thermal dissociation at 95 °C for 15 s, 60 °C for 60 s, and 95 °C for 1 s. Melting curve analysis of every qPCR was conducted after each cycle. The relative expression was calculated as previously described [81]. The fold change was calculated by expressing the target gene expression in the infected samples relative to the controls, normalized to GAPDH.

### 4.9. Histopathology and Immunofluorescence Assay

As previously published, 5 μm-thick paraffin sections of right cranial lung tissues were collected onto positively charged slides prior to hematoxylin and eosin (H&E) staining and immunofluorescence assay (IFA) [31]. H&E staining, microscopic evaluation, and histopathologic scoring of the lung tissues were carried out as previously published [31]. Briefly, the lung tissues were assigned a quantitative histopathological score from 0 to 4 (0 = no change, 1 = minimal change, 2 = mild change, 3 = moderate change, 4 = marked change) and scored by a blinded, board-certified veterinary pathologist. In IFA, slides were rehydrated using toluene and ethanol changes. Citrate unmasking solution (Cell Signal, Danvers, MA, USA) was then used for antigen retrieval. Slides were blocked with 10% goat serum for 1 h following overnight incubation with respective primary antibodies; MPO in 1:200 dilution (Thermofisher, Wilmington, DE, USA), neutrophil elastase in 1:200 dilution (NE: Invitrogen, Thermofisher, Wilmington, DE, USA), citrullinated histone H3 in 1:200 dilution (Cit. H3, Abbomax, CA, USA). Immunoreactivity was detected using anti-rabbit Alexa fluor 555 secondary antibody (Cell Signaling, Danvers, MA, USA) and 4′,6-diamidino-2-phenylindole (DAPI, Cell Signaling, Danvers, MA, USA) as the nuclear counterstain [82]. The slides were visualized using Zeiss LSM 980 Airyscan 2 Confocal Laser Scanning Microscope (Zeiss, White Plains, NY, USA) and images were retrieved and analyzed using ZEN blue software (v.1.10).

### 4.10. MPO-DNA ELISA

MPO-DNA complexes in both plasma and BALF were detected via MPO-DNA enzyme-linked immunosorbent assay (ELISA) which was adopted from previous literature [83]. In brief, a 96-well plate was coated with 100 μL of rabbit anti-myeloperoxidase polyclonal antibody (Thermofisher, Wilmington, DE, USA) diluted at 1:1000 and incubated overnight on a plate shaker at 4 °C. Following the washes, the plate was blocked with 1% bovine serum albumin (Millipore-Sigma, Burlington, MA, USA) in PBS and incubated for 2 h at room temperature. Plasma or BALF samples (20 μL) were mixed with peroxidase-labeled anti-DNA detection antibody (Cell Death Detection ELISA kit, Roche, Millipore-Sigma, Burlington, MA, USA) which was diluted 1:40 in the incubation buffer provided with the kit. A total volume of 100 μL was added to each well. The complexes were detected by adding 100 μL of 2,2′-azino-bis-(3-ethylbenzothiazoline-6-sulphonic acid (ABTS) as the substrate following the addition of ABTS stop solution, both provided with the kit. The optical density of each well was measured at 405 nm using the spectrometer and compared against the controls.

### 4.11. Quant-iT PicoGreen dsDNA Assay

The NET-derived DNA was measured using Quant-iT Pico Green dsDNA assay kit (Thermofisher, Wilmington, DE, USA), according to the manufacturer’s recommendations. Briefly, the experimental samples were diluted at a 1:1 ratio in TE buffer to a final volume of 100 μL in a 96-well plate following the addition of 100 μL of the aqueous working solution of the Quant-iT™ PicoGreen™ dsDNA Reagent to each sample. Sample fluorescence was measured using a fluorescence microplate reader (Cytation-5, Agilent, Santa Clara, CA, USA) at standard fluorescein wavelengths (excitation ∼480 nm, emission ∼520 nm).

### 4.12. Lung RNA Sequencing and Analysis

RNA sequencing was performed on the lungs of all cats as previously published [31]. Briefly, RNA was extracted from lungs using a QIAGEN RNA isolation kit (Thermofisher, Wilmington, DE, USA). cDNA library preparation and RNA sequencing was performed by Novogene Co Ltd. (Sacramento, CA, USA), generating paired-end reads at 150 bp length on an Illumina platform. The raw sequencing data reads were deposited under BioProject accession number PRJNA842733 in the NCBI Sequence Read Archive database (SRA) [31]. These sequences were downloaded and utilized for the current analysis. Briefly, the metadata were downloaded and converted to fastq format using a Linux command pipeline via the SRA toolkit [84]. The fastq files were then aligned with the *F.catus_Fca126_mat1.0_*genome sequence file [85] using STAR (v 2.7.3a) [86] with the default parameters and quantification mode of gene counts. Gene counts were utilized for differential expression analysis in R (v 4.3.2) using the package DESeq2 (v1.40.2) [87]. Differentially expressed genes (DEGs) were obtained for controls vs. 4 dpi, controls vs. 12 dpi, and 4 dpi vs. 12 dpi with Benjamini–Hochberg (BH) adjusted *p*-value < 0.05. The ggplot2 package (v 3.4.4.) [88] was used to create volcano plots that graphically represented the statistical outcomes of the comparisons. The plot was created using the negative logarithm (base 10) of the *p*-values on the y-axis and the log fold changes on the x-axis. Heatmaps were drawn to further elaborate the alterations in DEGs related to neutrophil activation and NETosis in each compared category. The DEGs from all comparisons (4 dpi vs controls, 12 dpi vs controls, 4 vs 12 dpi) were subjected to the Gene Ontology (GO) [89] and Kyoto Encyclopedia of Genes and Genomes (KEGG) pathway analysis [90] through the online DAVID bioinformatics tools (v 6.8) [91]. GO terms and KEGG pathways with BH-adjusted *p*-value < 0.05 were retained in the enrichment analysis. The bubble plots and bar plots for enrichment analysis were drawn for selected neutrophil activation and NETosis-specific pathways using the ggplot2 in R. Heatmaps were drawn to further elaborate the alterations in DEGs related to neutrophil activation and NETosis in each compared category.

### 4.13. Statistical Analyses

All statistical analyses were performed using GraphPad Prism (V10.1.2) [92]. Nonparametric data were represented as mean ± SEM. One-way or two-way ANOVA and post hoc Tukey’s test were used for multiple group comparisons. Correlations were calculated using Pearson coefficient correlation. A *p*-value of 0.05 or less was considered statistically significant.

## Data Availability

The data supporting the findings of this study are deposited at https://doi.org/10.5281/zenodo.13759699 (accessed on 13 September 2024).

## Supplementary Materials

The following supporting information can be downloaded at: https://www.mdpi.com/article/10.3390/ijms251810054/s1.

## References

[1] Lai C.-C., Shih T.-P., Ko W.-C., Tang H.-J., Hsueh P.-R. (2020). Severe acute respiratory syndrome coronavirus 2 (SARS-CoV-2) and coronavirus disease-2019 (COVID-19): The epidemic and the challenges. Int. J. Antimicrob. Agents.

[2] Aleem A., Akbar Samad A.B., Vaqar S. (2021). Emerging variants of SARS-CoV-2 and novel therapeutics against coronavirus (COVID-19). StatPearls.

[3] Cascella M., Rajnik M., Aleem A., Dulebohn S.C., Di Napoli R. (2020). Features, evaluation, and treatment of coronavirus (COVID-19). StatPearls.

[4] Stokol T., McAloose D., Terio K.A., Salguero F.J. (2020). Severe Acute Respiratory Syndrome-Coronavirus-2 (SARS-CoV-2): A Perspective Through the Lens of the Veterinary Diagnostic Laboratory. Front. Vet. Sci..

[5] Cesta M.C., Zippoli M., Marsiglia C., Gavioli E.M., Cremonesi G., Khan A., Mantelli F., Allegretti M., Balk R. (2023). Neutrophil activation and neutrophil extracellular traps (NETs) in COVID-19 ARDS and immunothrombosis. Eur. J. Immunol..

[6] Al-Anazi K., Al-Anazi W., Al-Jasser A. (2020). Neutrophils, NETs, NETosis and their paradoxical roles in COVID-19. J. Stem Cell Ther. Transplant..

[7] Kasuga Y., Zhu B., Jang K.-J., Yoo J.-S. (2021). Innate immune sensing of coronavirus and viral evasion strategies. Exp. Mol. Med..

[8] Mantovani A., Cassatella M.A., Costantini C., Jaillon S. (2011). Neutrophils in the activation and regulation of innate and adaptive immunity. Nat. Rev. Immunol..

[9] Ricci D., Etna M.P., Rizzo F., Sandini S., Severa M., Coccia E.M. (2021). Innate Immune Response to SARS-CoV-2 Infection: From Cells to Soluble Mediators. Int. J. Mol. Sci..

[10] Rudd J.M., Pulavendran S., Ashar H.K., Ritchey J.W., Snider T.A., Malayer J.R., Marie M., Chow V.T., Narasaraju T. (2019). Neutrophils induce a novel chemokine receptors repertoire during influenza pneumonia. Front. Cell. Infect. Microbiol..

[11] Ma Y., Zhang Y., Zhu L. (2021). Role of neutrophils in acute viral infection. Immun. Inflamm. Dis..

[12] Ashar H.K., Pulavendran S., Rudd J.M., Maram P., Achanta M., Chow V.T., Malayer J.R., Snider T.A., Teluguakula N. (2021). Administration of a CXC chemokine receptor 2 (CXCR2) antagonist, SCH527123, together with oseltamivir suppresses NETosis and protects mice from lethal influenza and piglets from swine-influenza infection. Am. J. Pathol..

[13] Barnes B.J., Adrover J.M., Baxter-Stoltzfus A., Borczuk A., Cools-Lartigue J., Crawford J.M., Daßler-Plenker J., Guerci P., Huynh C., Knight J.S. (2020). Targeting potential drivers of COVID-19: Neutrophil extracellular traps. J. Exp. Med..

[14] Liu J., Liu Y., Xiang P., Pu L., Xiong H., Li C., Zhang M., Tan J., Xu Y., Song R. (2020). Neutrophil-to-lymphocyte ratio predicts critical illness patients with 2019 coronavirus disease in the early stage. J. Transl. Med..

[15] Nappi F., Chello M., Bellomo F., Bellomo F., Chen X., Singh S.S.A. (2022). Insights into the Role of Neutrophils and Neutrophil Extracellular Traps in Causing Cardiovascular Complications in Patients with COVID-19: A Systematic Review. J. Clin. Med..

[16] Chan L., Karimi N., Morovati S., Alizadeh K., Kakish J.E., Vanderkamp S., Fazel F., Napoleoni C., Alizadeh K., Mehrani Y. (2021). The roles of neutrophils in cytokine storms. Viruses.

[17] Ackermann M., Anders H.-J., Bilyy R., Bowlin G.L., Daniel C., De Lorenzo R., Egeblad M., Henneck T., Hidalgo A., Hoffmann M. (2021). Patients with COVID-19: In the dark-NETs of neutrophils. Cell Death Differ..

[18] Obermayer A., Jakob L.-M., Haslbauer J.D., Matter M.S., Tzankov A., Stoiber W. (2021). Neutrophil Extracellular Traps in Fatal COVID-19-Associated Lung Injury. Dis. Markers.

[19] Ashar H.K., Mueller N.C., Rudd J.M., Snider T.A., Achanta M., Prasanthi M., Pulavendran S., Thomas P.G., Ramachandran A., Malayer J.R. (2018). The role of extracellular histones in influenza virus pathogenesis. Am. J. Pathol..

[20] Cicco S., Cicco G., Racanelli V., Vacca A. (2020). Neutrophil extracellular traps (NETs) and damage-associated molecular patterns (DAMPs): Two potential targets for COVID-19 treatment. Mediat. Inflamm..

[21] Zuo Y., Yalavarthi S., Shi H., Gockman K., Zuo M., Madison J.A., Blair C., Weber A., Barnes B.J., Egeblad M. (2020). Neutrophil extracellular traps (NETs) as markers of disease severity in COVID-19. medRxiv.

[22] Chen T., Li Y., Sun R., Hu H., Liu Y., Herrmann M., Zhao Y., Muñoz L.E. (2021). Receptor-mediated NETosis on neutrophils. Front. Immunol..

[23] Pandolfi L., Bozzini S., Frangipane V., Percivalle E., De Luigi A., Violatto M.B., Lopez G., Gabanti E., Carsana L., D’Amato M. (2021). Neutrophil Extracellular Traps Induce the Epithelial-Mesenchymal Transition: Implications in Post-COVID-19 Fibrosis. Front. Immunol..

[24] Jing H., Chen X., Zhang S., Liu H., Zhang C., Du J., Li Y., Wu X., Li M., Xiang M. (2021). Neutrophil extracellular traps (NETs): The role of inflammation and coagulation in COVID-19. Am. J. Transl. Res..

[25] Bi Z., Hong W., Yang J., Lu S., Peng X. (2021). Animal models for SARS-CoV-2 infection and pathology. MedComm.

[26] Jia W., Wang J., Sun B., Zhou J., Shi Y., Zhou Z. (2021). The Mechanisms and Animal Models of SARS-CoV-2 Infection. Front. Cell Dev. Biol..

[27] Gunasekara S., Selvan M.T., Miller C.A., Rudd J.M. (2022). Thinking Outside the Box: Utilizing Nontraditional Animal Models for COVID-19 Research. Int. J. Transl. Med..

[28] Bosco-Lauth A.M., Hartwig A.E., Porter S.M., Gordy P.W., Nehring M., Byas A.D., VandeWoude S., Ragan I.K., Maison R.M., Bowen R.A. (2020). Experimental infection of domestic dogs and cats with SARS-CoV-2: Pathogenesis, transmission, and response to reexposure in cats. Proc. Natl. Acad. Sci. USA.

[29] Shi J., Wen Z., Zhong G., Yang H., Wang C., Huang B., Liu R., He X., Shuai L., Sun Z. (2020). Susceptibility of ferrets, cats, dogs, and other domesticated animals to SARS–coronavirus 2. Science.

[30] Rudd J.M., Selvan M.T., Cowan S., Kao Y.-F., Midkiff C.C., Narayanan S., Ramachandran A., Ritchey J.W., Miller C.A. (2021). Clinical and Histopathologic Features of a Feline SARS-CoV-2 Infection Model Are Analogous to Acute COVID-19 in Humans. Viruses.

[31] Selvan M.T., Gunasekara S., Xiao P., Griffin K., Cowan S.R., Narayanan S., Ramachandran A., Hagen D.E., Ritchey J.W., Rudd J.M. (2022). SARS-CoV-2 (Delta variant) infection kinetics and immunopathogenesis in domestic cats. Viruses.

[32] Gu T., Zhao S., Jin G., Song M., Zhi Y., Zhao R., Ma F., Zheng Y., Wang K., Liu H. (2021). Cytokine signature induced by SARS-CoV-2 spike protein in a mouse model. Front. Immunol..

[33] Hsu R.-J., Yu W.-C., Peng G.-R., Ye C.-H., Hu S., Chong P.C.T., Yap K.Y., Lee J.Y.C., Lin W.C., Yu S.H. (2022). The role of cytokines and chemokines in severe acute respiratory syndrome coronavirus 2 infections. Front. Immunol..

[34] Noroozi R., Branicki W., Pyrc K., Łabaj P.P., Pospiech E., Taheri M., Ghafouri-Fard S. (2020). Altered cytokine levels and immune responses in patients with SARS-CoV-2 infection and related conditions. Cytokine.

[35] Winkler E.S., Bailey A.L., Kafai N.M., Nair S., McCune B.T., Yu J., Fox J.M., Chen R.E., Earnest J.T., Keeler S.P. (2020). SARS-CoV-2 infection of human ACE2-transgenic mice causes severe lung inflammation and impaired function. Nat. Immunol..

[36] Costela-Ruiz V.J., Illescas-Montes R., Puerta-Puerta J.M., Ruiz C., Melguizo-Rodríguez L. (2020). SARS-CoV-2 infection: The role of cytokines in COVID-19 disease. Cytokine Growth Factor Rev..

[37] Olbei M., Hautefort I., Modos D., Treveil A., Poletti M., Gul L., Shannon-Lowe C.D., Korcsmaros T. (2021). SARS-CoV-2 causes a different cytokine response compared to other cytokine storm-causing respiratory viruses in severely ill patients. Front. Immunol..

[38] Zheng M., Karki R., Williams E.P., Yang D., Fitzpatrick E., Vogel P., Jonsson C.B., Kanneganti T.D. (2021). TLR2 senses the SARS-CoV-2 envelope protein to produce inflammatory cytokines. Nat. Immunol..

[39] Chu Y.-K., Ali G.D., Jia F., Li Q., Kelvin D., Couch R.C., Harrod K.S., Hutt J.A., Cameron C., Weiss S.R. (2008). The SARS-CoV ferret model in an infection-challenge study. Virology.

[40] Chen Y., Wang J., Liu C., Su L., Zhang D., Fan J., Yang Y., Xiao M., Xie J., Xu Y. (2020). IP-10 and MCP-1 as biomarkers associated with disease severity of COVID-19. Mol. Med..

[41] Polidoro R.B., Hagan R.S., de Santis Santiago R., Schmidt N.W. (2020). Overview: Systemic inflammatory response derived from lung injury caused by SARS-CoV-2 infection explains severe outcomes in COVID-19. Front. Immunol..

[42] Macdonald M.E., Weathered R., Stewart E.C., Magold A.I., Mukherjee A., Gurbuxani S., Smith H., McMullen P., Mueller J., Husain A.N. (2022). Lymphatic coagulation and neutrophil extracellular traps in lung-draining lymph nodes of COVID-19 decedents. Blood Adv..

[43] Borges L., Pithon-Curi T.C., Curi R., Hatanaka E. (2020). COVID-19 and neutrophils: The relationship between hyperinflammation and neutrophil extracellular traps. Mediators Inflamm..

[44] Blanch-Ruiz M.A., Ortega-Luna R., Martínez-Cuesta M.Á., Álvarez Á. (2021). The neutrophil secretome as a crucial link between inflammation and thrombosis. Int. J. Mol. Sci..

[45] Li J.-P., Wu K.-H., Chao W.-R., Lee Y.-J., Yang S.-F., Chao Y.-H. (2022). Immunomodulation of mesenchymal stem cells in acute lung injury: From preclinical animal models to treatment of severe COVID-19. Int. J. Mol. Sci..

[46] Deng Q., Pan B., Alam H.B., Liang Y., Wu Z., Liu B., Mor-Vaknin N., Duan X., Williams A.M., Tian Y. (2020). Citrullinated histone H3 as a therapeutic target for endotoxic shock in mice. Front. Immunol..

[47] Pulavendran S., Prasanthi M., Ramachandran A., Grant R., Snider T.A., Chow V.T., Malayer J.R., Teluguakula N. (2020). Production of neutrophil extracellular traps contributes to the pathogenesis of Francisella tularemia. Front. Immunol..

[48] Romero V., Fert-Bober J., Nigrovic P.A., Darrah E., Haque U.J., Lee D.M., Van Eyk J., Rosen A., Andrade F. (2013). Immune-mediated pore-forming pathways induce cellular hypercitrullination and generate citrullinated autoantigens in rheumatoid arthritis. Sci. Transl. Med..

[49] Elkon K.B. (2018). Cell death, nucleic acids, and immunity: Inflammation beyond the grave. Arthritis Rheumatol..

[50] Hamam H.J., Palaniyar N. (2019). Post-translational modifications in NETosis and NETs-mediated diseases. Biomolecules.

[51] Gierlikowska B., Stachura A., Gierlikowski W., Demkow U. (2022). The Impact of Cytokines on Neutrophils’ Phagocytosis and NET Formation during Sepsis—A Review. Int. J. Mol. Sci..

[52] Arcanjo A., Logullo J., Menezes C.C.B., de Souza Carvalho Giangiarulo T.C., Reis M.C.D., de Castro G.M.M., da Silva Fontes Y., Todeschini A.R., Freire-de-Lima L., Decoté-Ricardo D. (2020). The emerging role of neutrophil extracellular traps in severe acute respiratory syndrome coronavirus 2 (COVID-19). Sci. Rep..

[53] Radermecker C., Detrembleur N., Guiot J., Cavalier E., Henket M., d’Emal C., Vanwinge C., Cataldo D., Oury C., Delvenne P. (2020). Neutrophil extracellular traps infiltrate the lung airway, interstitial, and vascular compartments in severe COVID-19. J. Exp. Med..

[54] D’Agnillo F., Walters K., Xiao Y., Sheng Z., Scherler K., Park J., Gygli S., Rosas L.A., Sadtler K., Kalish H. (2021). Lung epithelial and endothelial damage, loss of tissue repair, inhibition of fibrinolysis, and cellular senescence in fatal COVID-19. Sci. Transl. Med..

[55] Manda-Handzlik A., Demkow U. (2019). The brain entangled: The contribution of neutrophil extracellular traps to the diseases of the central nervous system. Cells.

[56] Manda-Handzlik A., Demkow U. (2015). Neutrophils: The role of oxidative and nitrosative stress in health and disease. Pulm. Infect..

[57] Tan C., Aziz M., Wang P. (2021). The vitals of NETs. J. Leukoc. Biol..

[58] Huang J., Hong W., Wan M., Zheng L. (2022). Molecular mechanisms and therapeutic target of NETosis in diseases. MedComm.

[59] Goggs R., Jeffery U., LeVine D.N., Li R.H. (2020). Neutrophil-extracellular traps, cell-free DNA, and immunothrombosis in companion animals: A review. Vet. Pathol..

[60] Szturmowicz M., Demkow U. (2021). Neutrophil extracellular traps (NETs) in severe SARS-CoV-2 lung disease. Int. J. Mol. Sci..

[61] Becker R.C. (2020). COVID-19-associated vasculitis and vasculopathy. J. Thromb. Thrombolysis.

[62] Zhou Y., Xu Z., Liu Z. (2022). Impact of neutrophil extracellular traps on thrombosis formation: New findings and future perspective. Front. Cell. Infect. Microbiol..

[63] Zuo Y., Zuo M., Yalavarthi S., Gockman K., Madison J.A., Shi H., Woodard W., Lezak S.P., Lugogo N.L., Knight J.S. (2021). Neutrophil extracellular traps and thrombosis in COVID-19. J. Thromb. Thrombolysis.

[64] González-Jiménez P., Méndez R., Latorre A., Mengot N., Piqueras M., Reyes S., Moscardó A., Alonso R., Amara-Elori I., Menéndez R. (2023). Endothelial Damage, Neutrophil Extracellular Traps and Platelet Activation in COVID-19 vs. Community-Acquired Pneumonia: A Case–Control Study. Int. J. Mol. Sci..

[65] Vassiliou A.G., Kotanidou A., Dimopoulou I., Orfanos S.E. (2020). Endothelial damage in acute respiratory distress syndrome. Int. J. Mol. Sci..

[66] Demkow U. (2023). Molecular mechanisms of neutrophil extracellular trap (NETs) degradation. Int. J. Mol. Sci..

[67] Bai X., Hippensteel J., Leavitt A., Maloney J.P., Beckham D., Garcia C., Li Q., Freed B.M., Ordway D., Sandhaus R.A. (2021). Hypothesis: Alpha-1-antitrypsin is a promising treatment option for COVID-19. Med. Hypotheses.

[68] Lee Y.Y., Park H.H., Park W., Kim H., Jang J.G., Hong K.S., Lee J.Y., Seo H.S., Na D.H., Kim T.H. (2021). Long-acting nanoparticulate DNase-1 for effective suppression of SARS-CoV-2-mediated neutrophil activities and cytokine storm. Biomaterials.

[69] He Y., Yang F.-Y., Sun E.-W. (2018). Neutrophil extracellular traps in autoimmune diseases. Chin. Med. J..

[70] Mutua V., Gershwin L.J. (2021). A review of neutrophil extracellular traps (NETs) in disease: Potential anti-NETs therapeutics. Clin. Rev. Allergy Immunol..

[71] Bruschi M., Moroni G., Sinico R.A., Franceschini F., Fredi M., Vaglio A., Cavagna L., Petretto A., Pratesi F., Migliorini P. (2021). Neutrophil extracellular traps in the autoimmunity context. Front. Med..

[72] Barré-Sinoussi F., Montagutelli X. (2015). Animal models are essential to biological research: Issues and perspectives. Future Sci. OA.

[73] Di Guardo G. (2020). Giovanni Di Guardo: Animal Models and Pathogenetic Insights to COVID-19. J. Comp. Pathol..

[74] Lj R. (1938). A simple method of estimating fifty per cent endpoints. Am. J. Hyg..

[75] PW T. (1992). Measuring cytokine levels in blood. Importance of anticoagulants, processing, storing. J. Immunol. Methods.

[76] Gorman M.J., Patel N., Guebre-Xabier M., Zhu A.L., Atyeo C., Pullen K.M., Loos C., Goez-Gazi Y., Carrion R., Tian J.H. (2021). Fab and Fc contribute to maximal protection against SARS-CoV-2 following NVX-CoV2373 subunit vaccine with Matrix-M vaccination. Cell Rep. Med..

[77] Van Hoecke L., Job E.R., Saelens X., Roose K. (2017). Bronchoalveolar lavage of murine lungs to analyze inflammatory cell infiltration. J. Vis. Exp..

[78] Jewell D.E., Panickar K.S. (2021). Botanicals Reduce Circulating Concentrations of Cholesterol and Triglycerides and Work Synergistically With Arachidonic Acid to Reduce Inflammatory Cytokines in Cats. Front. Vet. Sci..

[79] Amirouche A., Ait-Ali D., Nouri H., Boudrahme-Hannou L., Tliba S., Ghidouche A., Bitam I. (2021). TRIzol-based RNA extraction for detection protocol for SARS-CoV-2 of coronavirus disease 2019. New Microbes New Infect..

[80] Hummon A.B., Lim S.R., Difilippantonio M.J., Ried T. (2007). Isolation and solubilization of proteins after TRIzol^®^ extraction of RNA and DNA from patient material following prolonged storage. Biotechniques.

[81] Rao X., Huang X., Zhou Z., Lin X. (2013). An improvement of the 2ˆ(−delta delta CT) method for quantitative real-time polymerase chain reaction data analysis. Biostat. Bioinforma. Biomath..

[82] Bradford M.M. (1976). A Rapid and Sensitive Method for the Quantitation of Microgram Quantities of Protein Utilizing the Principle of Protein-Dye Binding. Anal. Biochem..

[83] Hayden H., Ibrahim N., Klopf J., Zagrapan B., Mauracher L.-M., Hell L., Hofbauer T.M., Ondracek A.S., Schoergenhofer C., Jilma B. (2021). ELISA detection of MPO-DNA complexes in human plasma is error-prone and yields limited information on neutrophil extracellular traps formed in vivo. PLoS ONE.

[84] Leinonen R., Sugawara H., Shumway M., On behalf of the International Nucleotide Sequence Database Collaboration (2010). The sequence read archive. Nucleic Acids Res..

[85] Lopez J.V., Cevario S., O’Brien S.J. (1996). Complete nucleotide sequences of the domestic cat (*Felis catus*) mitochondrial genome and a transposed mtDNA tandem repeat (Numt) in the nuclear genome. Genomics.

[86] Dobin A., Davis C.A., Schlesinger F., Drenkow J., Zaleski C., Jha S., Batut P., Chaisson M., Gingeras T.R. (2013). STAR: Ultrafast universal RNA-seq aligner. Bioinformatics.

[87] Love M.I., Huber W., Anders S. (2014). Moderated estimation of fold change and dispersion for RNA-seq data with DESeq2. Genome Biol..

[88] Wickham H. (2016). Data Analysis. ggplot2.

[89] Harris M.A., Clark J., Ireland A., Lomax J., Ashburner M., Foulger R., Eilbeck K., Lewis S., Marshall B., Gene Ontology Consortium (2004). The Gene Ontology (GO) database and informatics resource. Nucleic Acids Res..

[90] Kanehisa M., Furumichi M., Tanabe M., Sato Y., Morishima K. (2017). KEGG: New perspectives on genomes, pathways, diseases and drugs. Nucleic Acids Res..

[91] Huang D.W., Sherman B.T., Lempicki R.A. (2009). Bioinformatics enrichment tools: Paths toward the comprehensive functional analysis of large gene lists. Nucleic Acids Res..

[92] Swift M.L. (1997). GraphPad Prism, Data Analysis, and Scientific Graphing. J. Chem. Inf. Comput. Sci..

