# Supplementary material for: Characterization of Neutrophil Functional Responses to SARS-CoV-2 Infection in a Translational Feline Model for COVID-19
Source: Int J Mol Sci. 2024 Sep 19;25(18):10054. doi: 10.3390/ijms251810054 (PMC11432149; doi:10.3390/ijms251810054)
Supplement: Supplementary file 1 [file ijms-25-10054-s001.zip › ijms-3196444-supplementary.pdf]

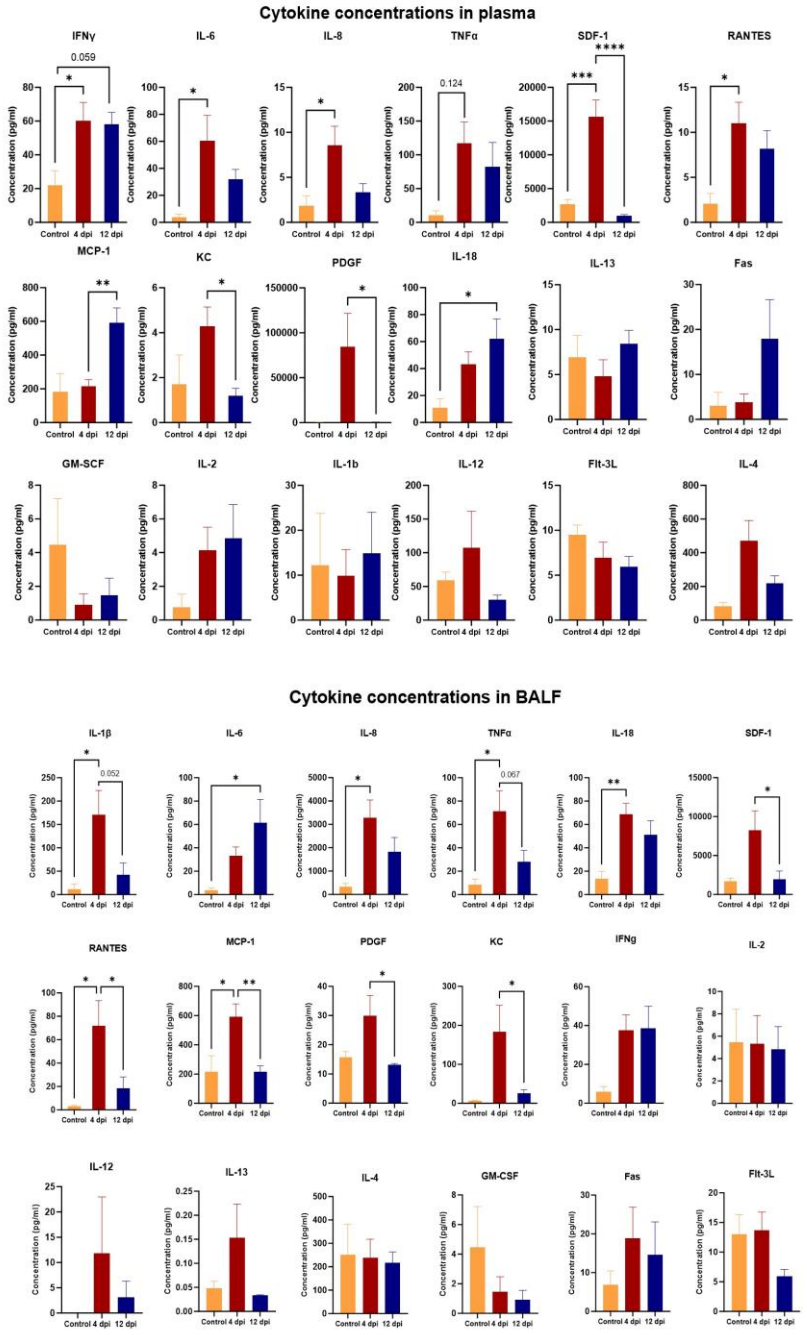

Figure S1: Analyzed cytokine concentrations in plasma and BALF from SARS-CoV-2 infected cats.

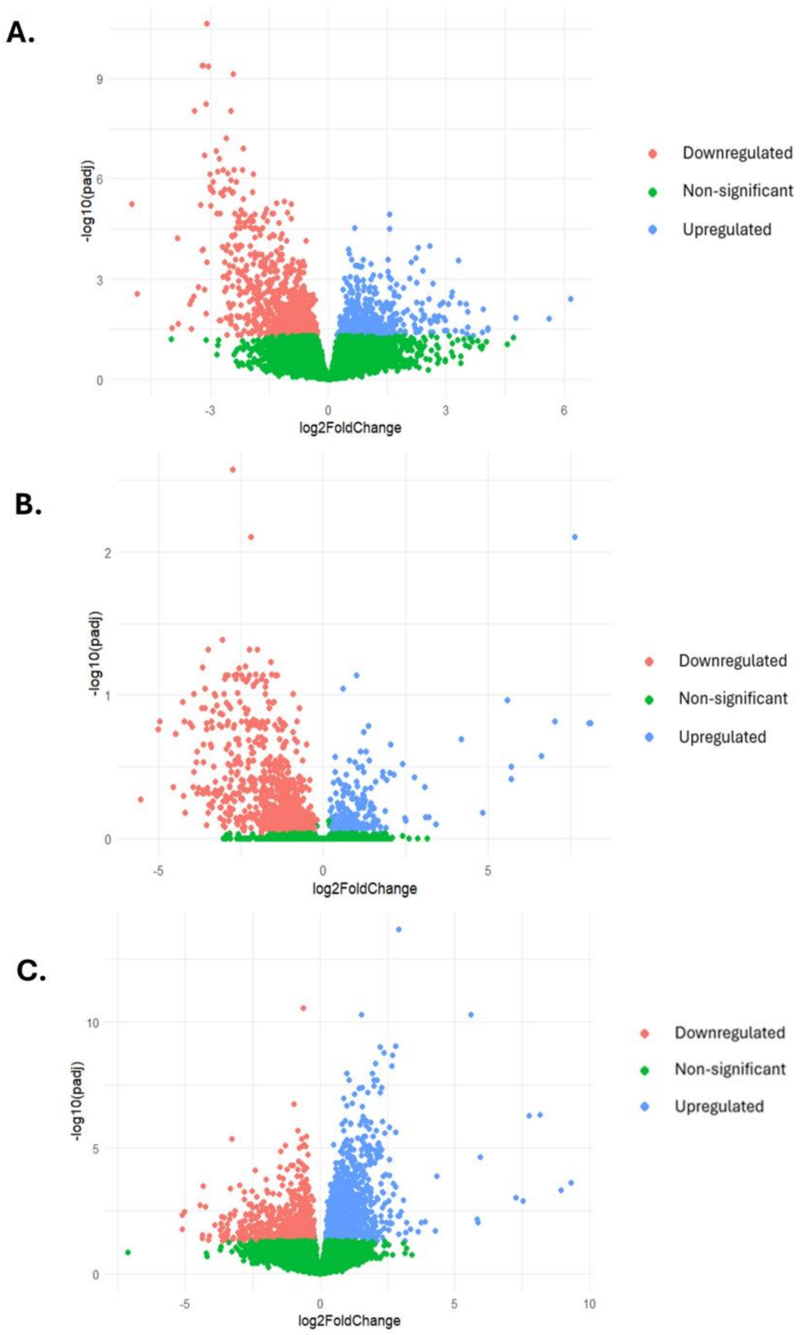

Figure S2: Differentially expressed genes (DEGs) in controls and SARS-CoV-2 infected cats. (A) Differentially expressed genes following comparisons between SARS-CoV-2 infected 4 dpi cats and sham-inoculated controls. The upregulated genes at 4dpi are shown in blue, while the downregulated genes at 4 dpi are highlighted in red. (B) Differentially expressed genes following comparisons between SARS-CoV-2 infected 12 dpi cats and sham-inoculated controls. The upregulated genes at 12 dpi are shown in blue and the downregulated genes are highlighted in red. (C) DEGs following comparisons between SARS-CoV-2 infected 4 dpi and 12 dpi cats. Upregulated genes at 4 dpi are shown in blue and downregulated genes at 4 dpi are highlighted in red.

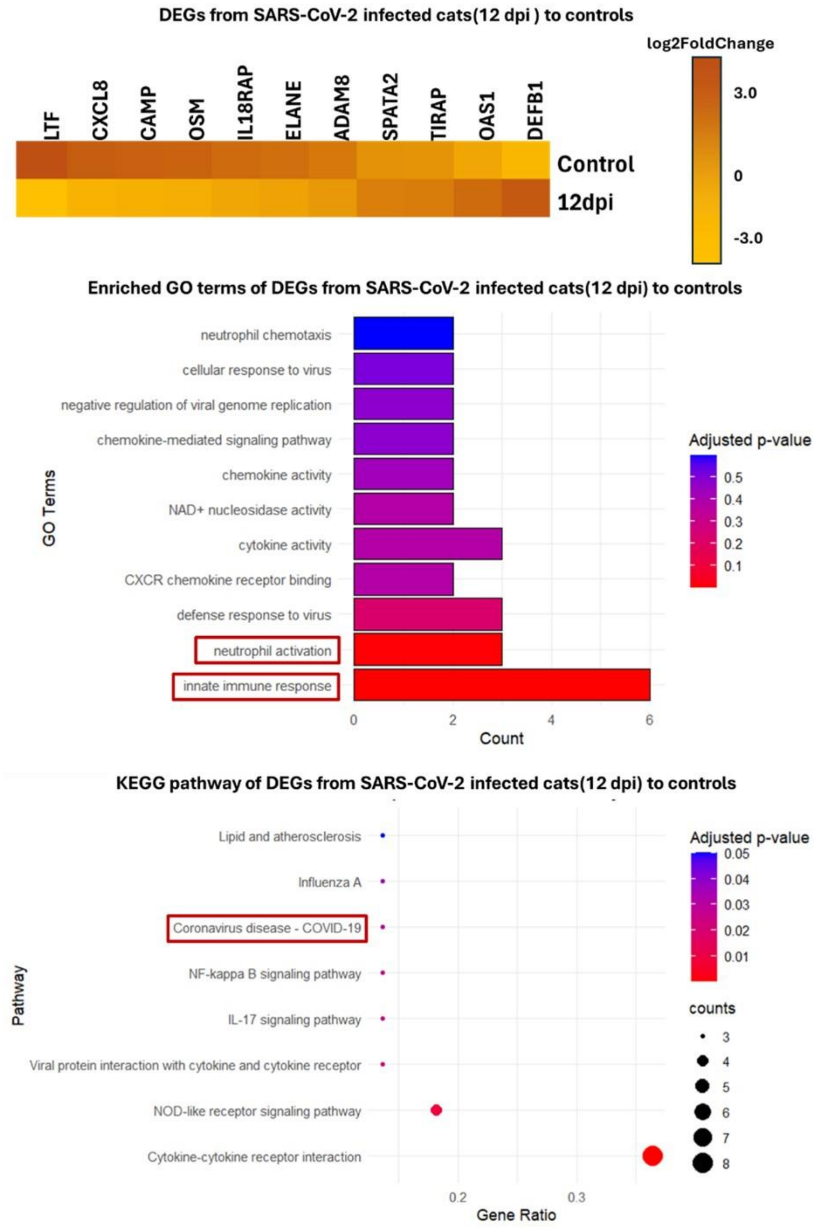

Figure S3: Heatmap and enrichment analysis of differentially expressed genes at 12 dpi SARS-CoV-2 infected cats versus sham-inoculated controls.

Table S1: Primer sets for real-time PCR.

Target	Primers
GAPDH	FW : 5'- AATTCCACGGCACAGTCAAGG-3'<br>RV : 5'- CATTTGATGTTGGCGGGATC-3'
NE	FW : 5'- CCAGCGGGTCTTTGAAAACG-3'<br>RV : 5'- ACCCGTACGTTGCTGTTGAT-3'
MPO	FW : 5'- CCC AGT TCA GGA AGC TCC G -3'<br>RV : 5'- CTT GGT CAG AAC AGG GAC GG -3'
Histone 3 (*H3*)	FW : 5'- TCGCTTTTGCTATGGCTCGT-3'<br>RV : 5'- GCATGATAGTGACGCGCTTG-3'
